# Post-Cracking Flexural Performance of HTPP Fiber-Reinforced EPS Concrete and Its Application to Wind-Loaded Exterior Wall Panels

**DOI:** 10.3390/ma19153287

**Published:** 2026-08-03

**Authors:** Huadong Cui, Yuhao Shang, Hanying Shou, Leiting Ye, Ji Yuan, Peixuan He, Ruige Li, Haijie He, Zihang Ding, Ziyu Mao

**Affiliations:** 1 Taizhou Construction Engineering Quality and Safety Affairs Center, Taizhou 318000, China; 2 School of Civil Engineering and Architecture, Taizhou University, Taizhou 318000, China

**Keywords:** HTPP fiber, expanded polystyrene concrete, post-cracking behavior, flexural toughness, exterior wall panel, wind load

## Abstract

Expanded polystyrene concrete (EPSC) offers low density and thermal insulation but is limited by brittle flexural failure. This study evaluates whether high-toughness modified polypropylene (HTPP) fibers can improve the post-cracking response of EPSC and translate the material-level gains into exterior wall-panel design under wind loading. Six mixtures containing 0, 0.3, 0.6, 0.9, 1.2, and 1.5 vol% HTPP fibers were prepared. Compressive tests, four-point bending tests, and scanning electron microscopy were conducted. Flexural toughness was assessed using average residual flexural strength together with the Nemkumar, literature-based, JSCE-SF4, and modified PCER methods. The mechanical indices generally increased and then declined as fiber content rose, with the best overall performance at 0.9 vol%. Relative to plain EPSC, this mixture increased compressive strength by 21.5%, flexural strength by 277.2%, and average residual flexural strength by 681.3%. The four toughness evaluation approaches produced consistent trends and showed that HTPP fibers most strongly improved post-peak load retention and energy absorption. Microscopic observations indicated that fiber bridging, crack-path deflection, stress transfer, and interfacial energy dissipation contributed to the enhanced toughness, whereas excessive fiber addition reduced the benefits. Using the measured residual capacity in a wind-load design model showed that HTPP fiber reinforcement can enlarge the allowable unsupported span of EPSC exterior wall panels. These findings identify an effective fiber dosage and provide a basis for the structural use of lightweight EPSC panels.

## 1. Introduction

EPS concrete (EPSC) is a lightweight thermal insulation concrete material with good thermal insulation performance. Due to its light weight, convenient construction and wide application in building components such as floor slabs, roof slabs and roof covers, it can effectively reduce building energy consumption [1,2,3]. However, EPSC usually has problems such as brittleness, low strength and easy cracking, which reduce the safety and durability of the structure to a certain extent [4,5]. One effective way to solve this problem is to add different types and scales of fibers to concrete to improve its load-bearing capacity after cracking and toughness [6].

Polypropylene (PP) fiber is the most widely used lightweight concrete reinforcement fiber at present. It can inhibit crack propagation and improve load-bearing capacity, but its tensile strength and toughness are limited, and it is still difficult to significantly improve the toughness of concrete under large deformation or high stress conditions [7,8]. In recent years, high-toughness polypropylene modified fibers [9] with higher tensile strength and elastic modulus have been proposed to enhance the post-cracking performance of concrete. For example, Lin et al. [10] found that HTPP fibers can significantly improve the post-cracking load-bearing capacity of lightweight concrete by bridging cracks; Lin et al. [11] studied the effect of fiber volume fraction on the post-cracking deformation performance of lightweight concrete, indicating that an appropriate amount of fiber can improve the toughness after cracking. However, at present, there are few studies on the toughening mechanism of HTPP fibers in EPS concrete. Currently, much research focuses on early crack initiation or a single mechanical property [12,13,14], lacking systematic research on enhancing the compressive strength and flexural toughness of EPSC.

Although existing studies have shown that HTPP fibers can improve the post-cracking load-bearing capacity and post-cracking deformation performance of concrete, the toughness of concrete is a multi-dimensional performance index, and a single mechanical parameter cannot fully reflect the toughening effect of fibers. Therefore, the quantitative evaluation of toughness is the key to further study of the toughening mechanism of HTPP fiber reinforced materials. To this end, scholars have proposed a variety of flexural toughness evaluation methods based on load-deflection curves, which can systematically study the effect of HTPP fibers on the toughness of EPS concrete [15]. In terms of flexural toughness evaluation methods, common flexural toughness evaluation methods for concrete can refer to ASTM C1018 [15], JSCE-SF4 [16], RILEM TC 162-TDF [17] and CECS13:2009 [18], etc. These methods are based on load-deflection curves to calculate the flexural toughness index of concrete. Although ASTM C1018 and CECS13:2009 can comprehensively evaluate the toughness of concrete, they rely on the initial crack deflection, which is prone to large errors. Therefore, adopting multiple methods for a comprehensive evaluation of toughness is helpful to analyze the toughness characteristics of concrete from different perspectives and improve the credibility and operability of the evaluation results.

Based on the above research status, this paper studies the toughening effect of HTPP fibers on the flexural toughness of EPS concrete by preparing EPS concrete specimens with different fiber volume fractions, carrying out four-point bending tests and various flexural toughness evaluations, and using the Nemkumar method, JSCE-SF4 method, literature analysis method, PCER and other methods to quantitatively analyze the toughness. Combined with scanning electron microscopy (SEM), the distribution and fracture morphology of fibers in EPSC are observed, and the micro-mechanism and toughening mechanism of HTPP fibers are revealed. Meanwhile, the ARS was adopted as the core evaluation index to quantify the HTPP fiber bridging effect and the flexural toughness of the material. Based on the experimental data, a calculation model for the spacing between support points of EPSC exterior wall panels under wind loads was established, thereby transforming the toughness properties at the material level into key parameters that can directly guide engineering design. This study can provide an experimental basis and theoretical guidance for the toughness optimization design and engineering application of lightweight concrete, and has important innovation and engineering application value.

## 2. Experimental Design

### 2.1. Experimental Materials

**Figure 1:**
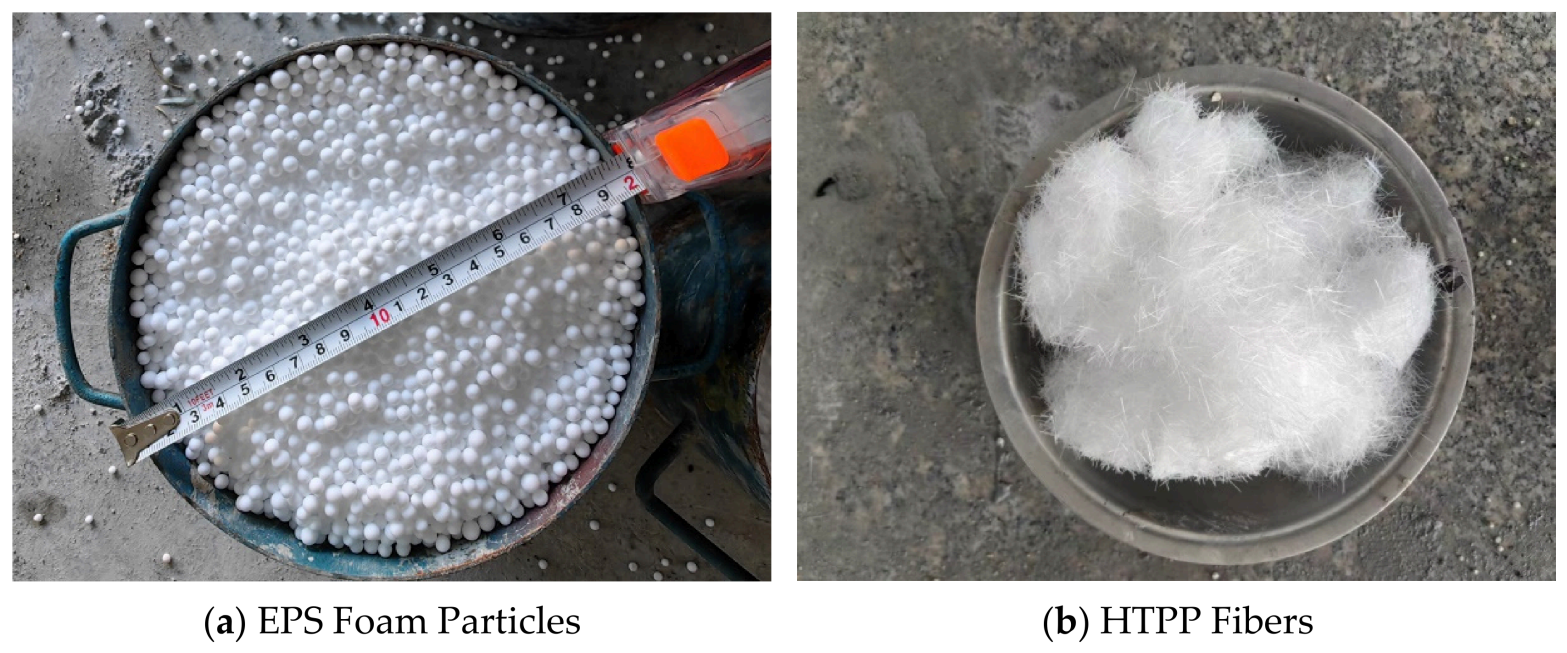
Raw materials.

The cement used in this study was P.O 42.5 cement produced by Anhui Conch Cement Co., Ltd. (Wuhu, China); Tap water was used as the mixing water. Class II fly ash was used as the mineral admixture. The fine aggregate was washed sea sand with a fineness modulus of 2.6. The water-reducing agent was a polycarboxylate superplasticizer (PCE) produced by Jiangsu Sobute New Materials Co., Ltd. (Nanjing, China), with a solid content of 30%. The EPS particles had a particle size of 4–6 mm and a bulk density of 4.8 kg/m^3^. The HTPP fibers were manufactured by Ningbo Shike New Materials Technology Co., Ltd. (Ningbo, China), and their properties are listed in Table 1. The EPS foam particles and HTPP fibers are shown in Figure 1.

### 2.2. Specimen Preparation

Six groups of EPS concrete specimens were designed in this study, with HTPP fiber volume contents of 0.0%, 0.3%, 0.6%, 0.9%, 1.2%, and 1.5%, respectively, while all other materials remained unchanged. The mix proportions of the six groups are listed in Table 2.

**Figure 2:**
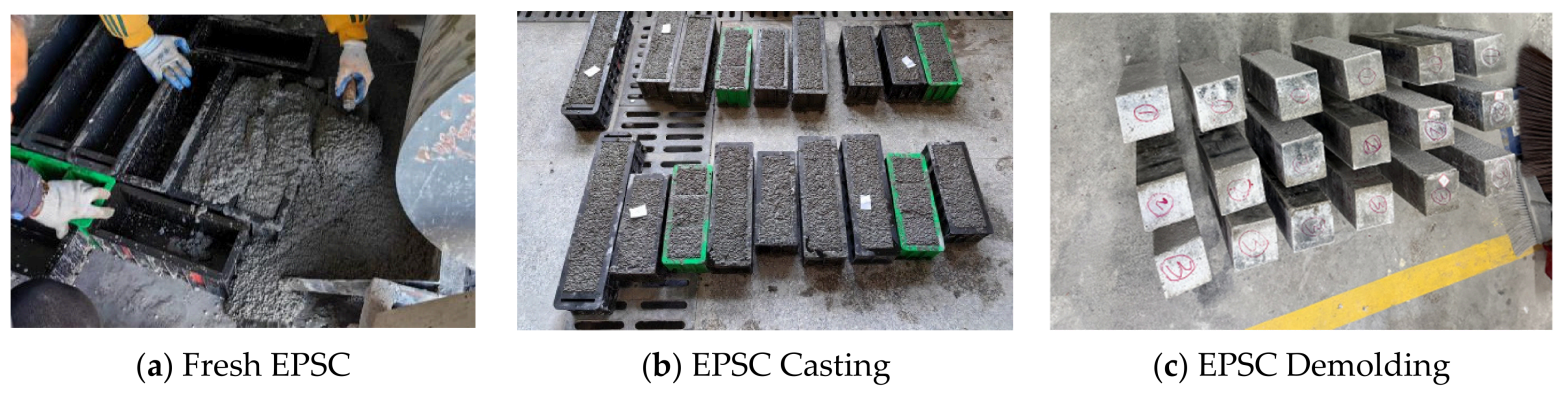
Preparation of EPSC specimens.

To ensure the uniform dispersion of fibers in the concrete matrix, a forced mixer was used for mixing, and the total mixing time was controlled at 3 min. The mixing sequence was as follows: first, the cementitious materials and sand were mixed for 60 s; then, the fibers were added and mixed for another 60 s; finally, water, admixture, and EPS particles were added and mixed for a further 60 s until a uniform mixture was obtained. After mixing, the fresh concrete was poured into molds. For each group, three specimens with dimensions of 100 × 100 × 100 mm were prepared for compressive strength testing, and three specimens with dimensions of 100 × 100 × 400 mm were prepared for flexural strength testing. The specimens were demolded after standing for 1 day and then transferred to a standard curing room for concrete. The curing temperature was maintained at 20 ± 2 °C. When the specimens reached the specified curing age (e.g., 28 days), they were removed for the corresponding mechanical tests. The specimen preparation process is shown in Figure 2.

### 2.3. Test Methods

#### 2.3.1. Mechanical Property Tests

**Figure 3:**
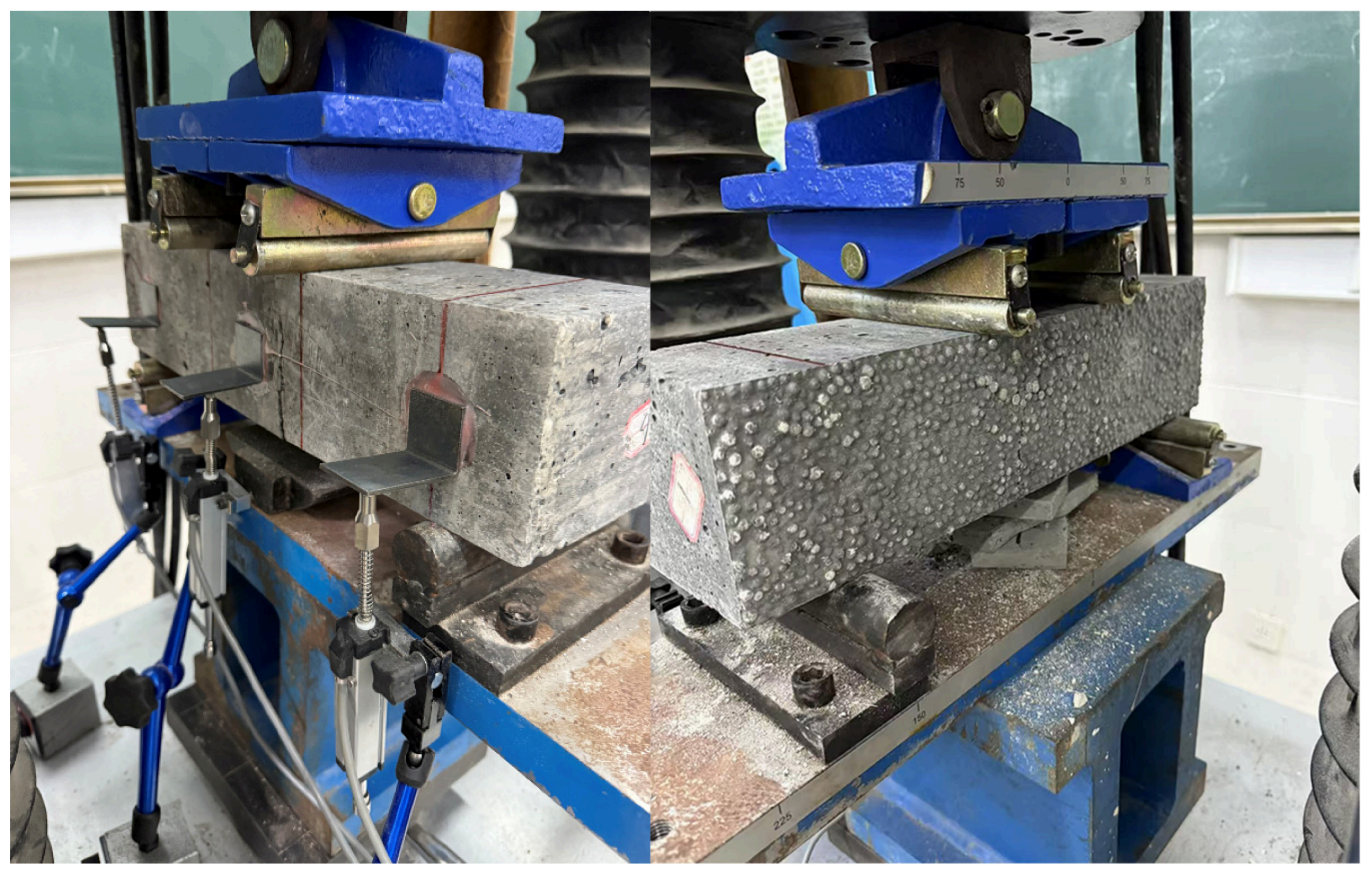
Schematic diagram of the flexural toughness test setup.

In this study, a universal testing machine was used to evaluate the flexural and compressive properties of EPSC. The flexural test was conducted in accordance with CECS 13:2009 [18], using a four-point bending loading method. The test setup is shown in Figure 3. Three L-shaped steel plates were attached to the center and fabricated positions on the side surface of each specimen, and LVDTs were installed beneath the steel plates to measure displacement. The mid-span displacement and support displacement were recorded, respectively, in order to obtain the load-displacement curves of the specimens. The deflection in the load-deflection curve was determined by subtracting the support displacement from the mid-span displacement. The compressive test was carried out using a 2000 kN servo testing machine at a loading rate of 0.2 mm/min until specimen failure occurred. All test results presented in this paper, including compressive strength, compressive strength retention, load-deflection curves, and peak flexural load, are the average values of three specimens.

#### 2.3.2. Microscopic Tests

The micro-morphology of the specimens was observed using scanning electron microscopy (SEM), and typical micrographs were recorded. The samples used for microscopic analysis were taken from the fractured EPSC debris after the mechanical tests. Three representative specimens were selected, and all samples were collected from the core region in the middle of the specimens to minimize the influence of edge effects on the microstructural characterization results. Relatively flat fragments were selected and processed into flake-like samples with a thickness of approximately 2 mm and dimensions of about 5–10 mm. Before testing, the samples were vacuum-dried and then sputter-coated with gold to improve their electrical conductivity and ensure imaging quality.

During the SEM test, the magnification range was 100–50,000×. Based on the observation of the specimen micro-morphology, the analysis focused on the distribution of HTPP fibers in the EPSC matrix, the interfacial bonding characteristics between the fibers and the cementitious materials, the morphology of the contact zone between EPS particles and the cementitious materials, and the characteristics of the gel products and pore structure within the matrix, in order to reveal the reinforcing and toughening mechanisms of HTPP fibers in EPSC.

## 3. Experimental Results

### 3.1. EPSC Mechanical Properties

#### 3.1.1. EPSC Compressive Strength

**Figure 4:**
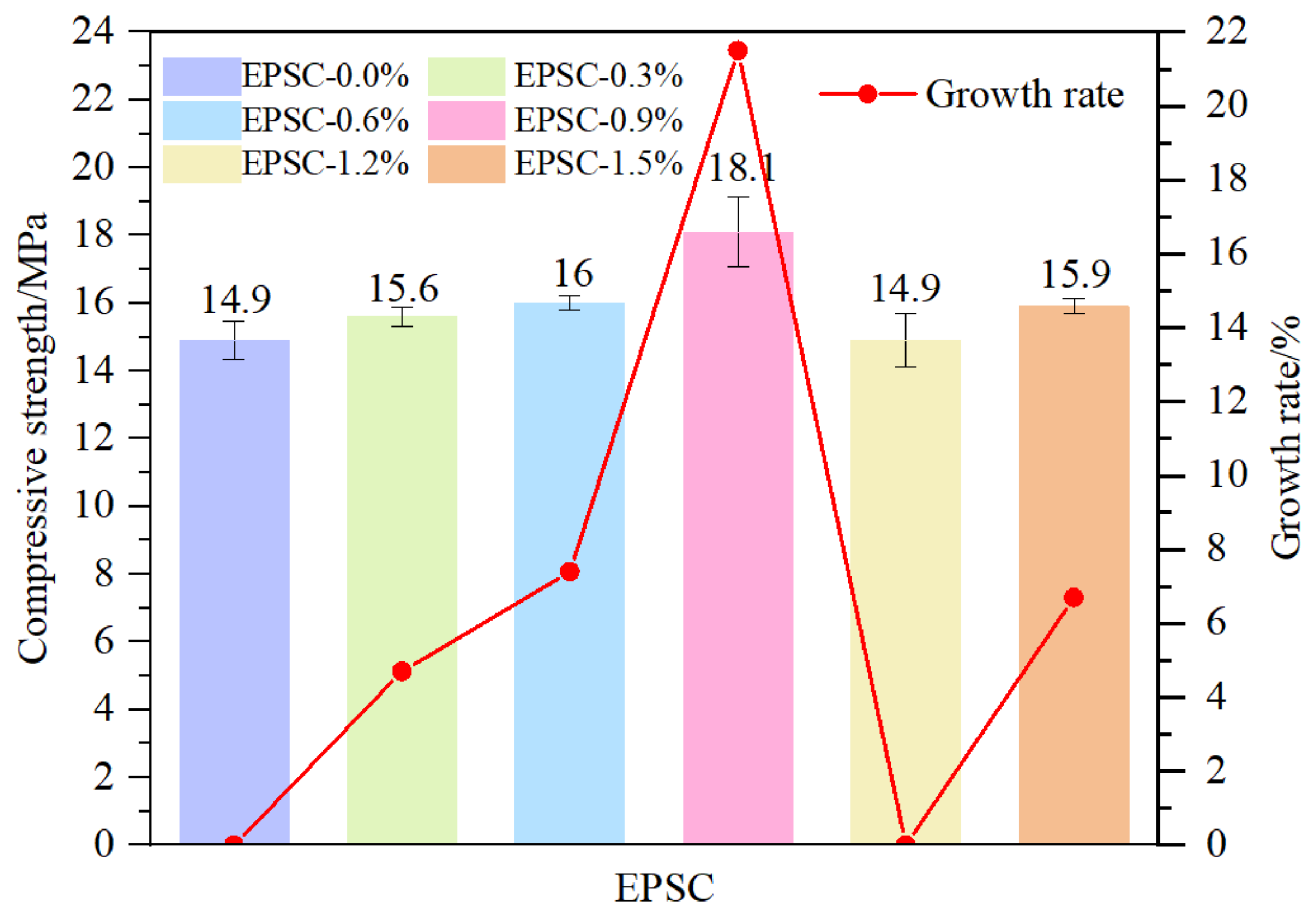
EPSC compressive strength of cube and its growth rate.

The cube compressive strength and growth rate curves of each EPSC group are shown in Figure 4. The cube compressive strength of EPSC first increases and then decreases with the increase of HTPP fiber content. The EPSC with 0.9% HTPP fiber exhibits the highest compressive strength. Compared with EPSC without HTPP fiber, the compressive strength of EPSC with HTPP fiber can be increased by up to 21.5%. As the HTPP fiber content continues to increase, the compressive strength of EPSC decreases instead. Notably, the strength of EPSC-1.2% is lower than that of EPSC-1.5%, which may be attributed to errors in specimen fabrication.

#### 3.1.2. EPSC Flexural Strength

**Figure 5:**
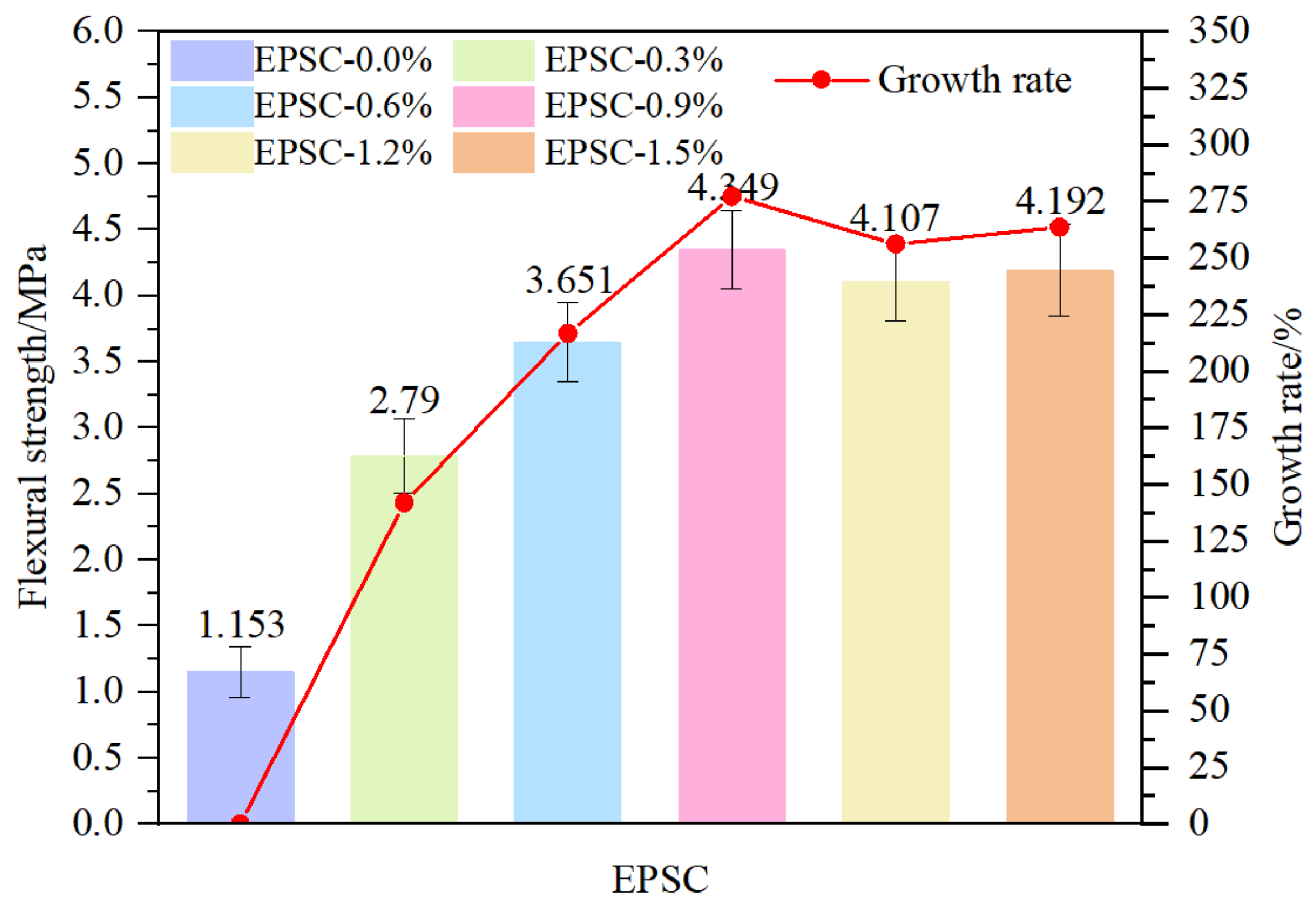
EPSC flexural strength and growth rate.

The flexural strength and growth rate curves of each EPSC group are shown in Figure 5. The flexural strength of EPSC first increases and then decreases with the addition of HTPP fibers. The EPSC with 0.9% HTPP fiber content exhibits the highest flexural strength. Compared with EPSC without HTPP fibers, the flexural strength of EPSC with HTPP fibers can be increased by up to 277.2%.

### 3.2. Flexural Failure Mode

**Figure 6:**
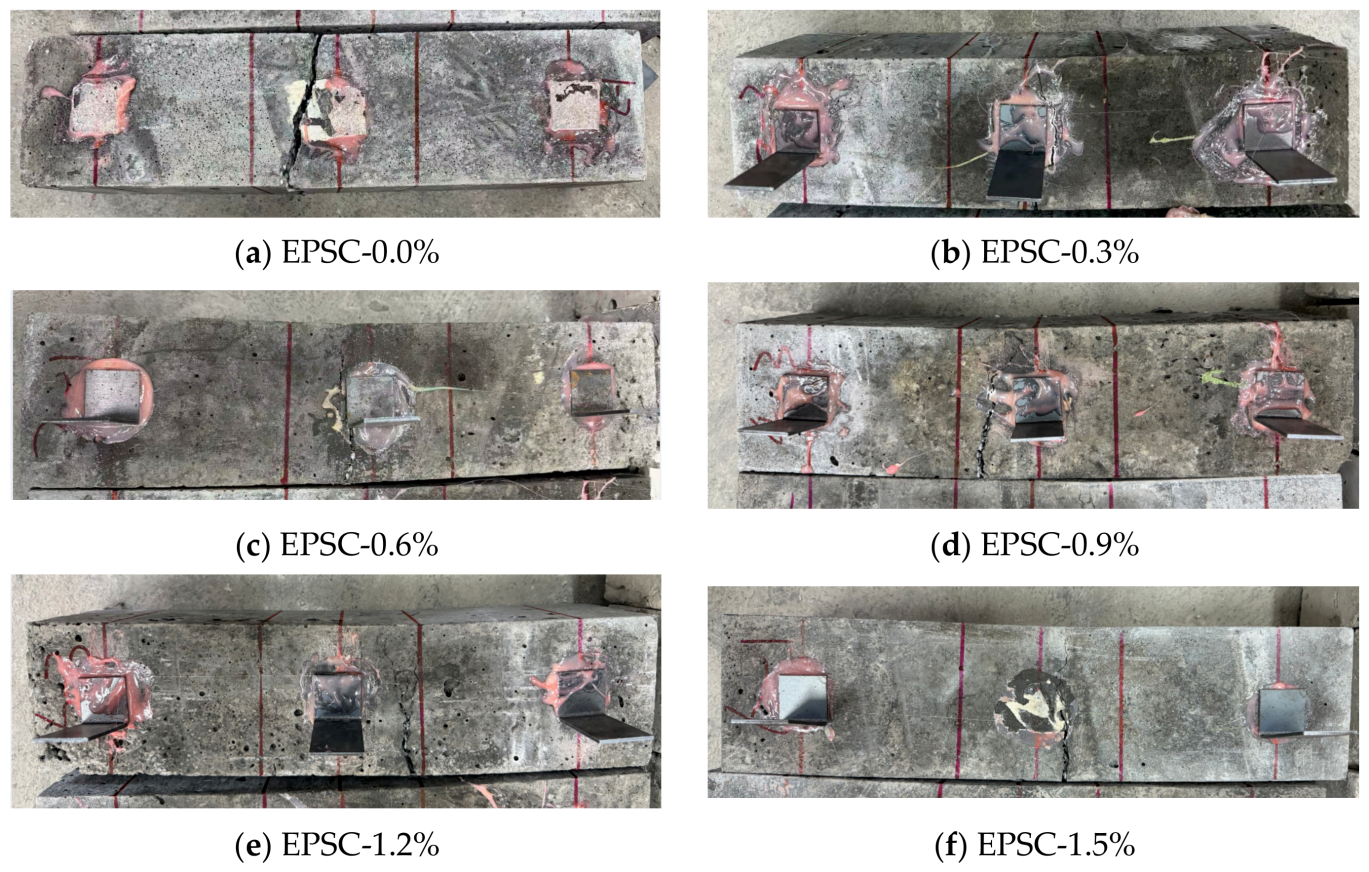
EPSC failure mode.

By conducting flexural tests on EPSC specimens with different HTPP fiber contents, several typical flexural failure modes were obtained, as shown in Figure 6. For the specimen without HTPP fibers, as the load increased, the cracks at the bottom of the specimen rapidly penetrated and suddenly fractured, leading to an immediate loss of load-bearing capacity. The main crack propagated in a nearly straight line, exhibiting obvious brittle failure characteristics, as illustrated in Figure 6a.

After the incorporation of HTPP fibers, the crack propagation path in EPSC specimens becomes significantly more tortuous, and the specimens exhibit a certain load-bearing capacity during the failure process. With an increase in HTPP fiber content, the trajectory of the main crack becomes further complicated, the number of micro-cracks increases, and some specimens even exhibit discontinuous cracks. As the load continues to increase, the fibers on both sides of the cracks are gradually straightened and begin to bear tensile stress, thereby effectively delaying crack propagation and extending the load-bearing duration before specimen failure. At the same time, the formation of multiple cracks alleviates stress concentration at the main crack, enhancing the crack resistance and overall toughness of the specimens. These effects demonstrate the positive role of HTPP fibers in improving the flexural strength of EPSC, as shown in Figure 6b–f.

Furthermore, as the HTPP fiber content further increases, when the fiber volume fraction exceeds 0.6%, the specimen still exhibits a relatively pronounced bending deformation state after failure, and the failed specimen shows a certain curvature, indicating that HTPP fibers can significantly enhance the toughness and deformability of EPSC.

### 3.3. Load-Deflection Curves

**Figure 7:**
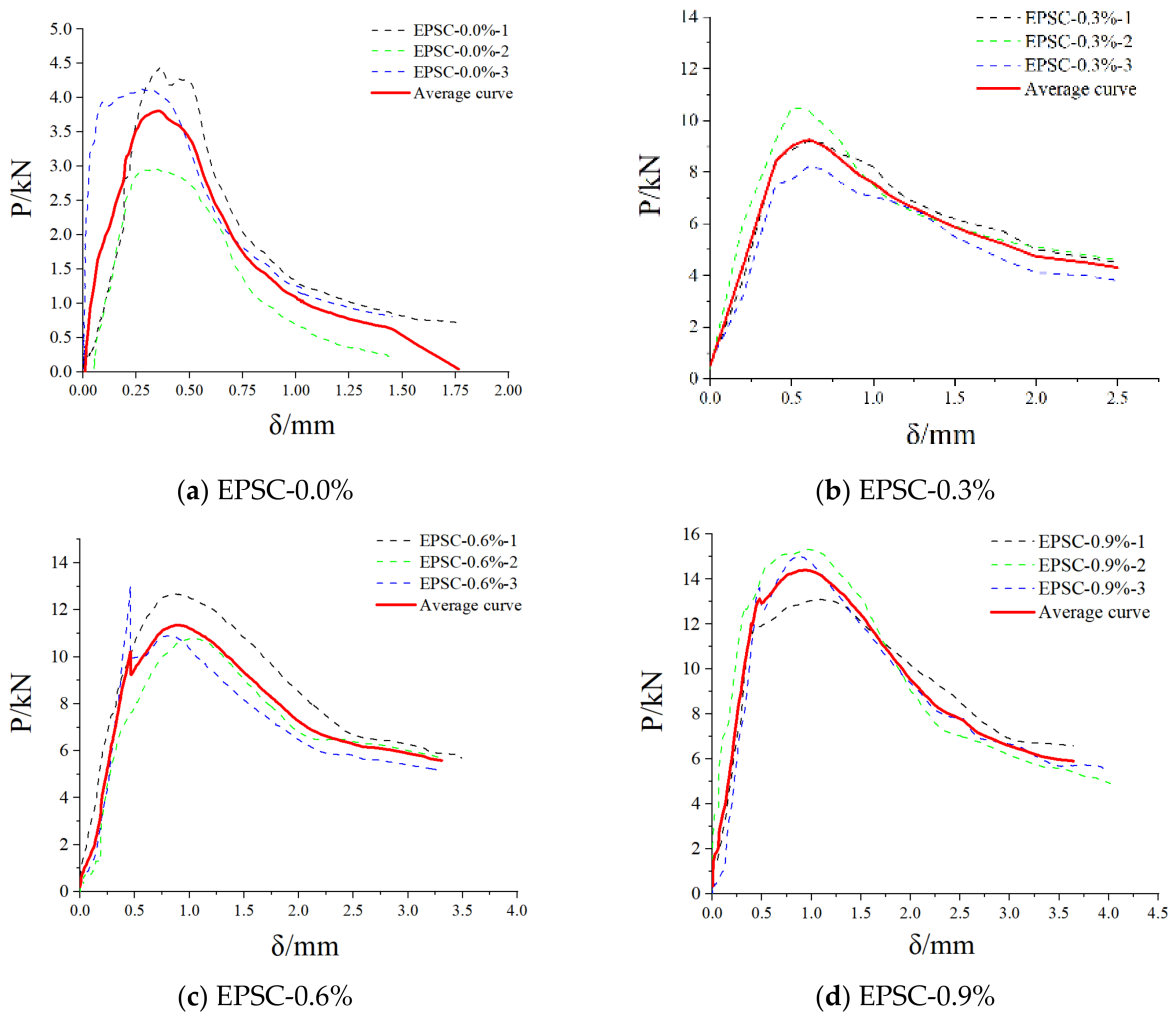

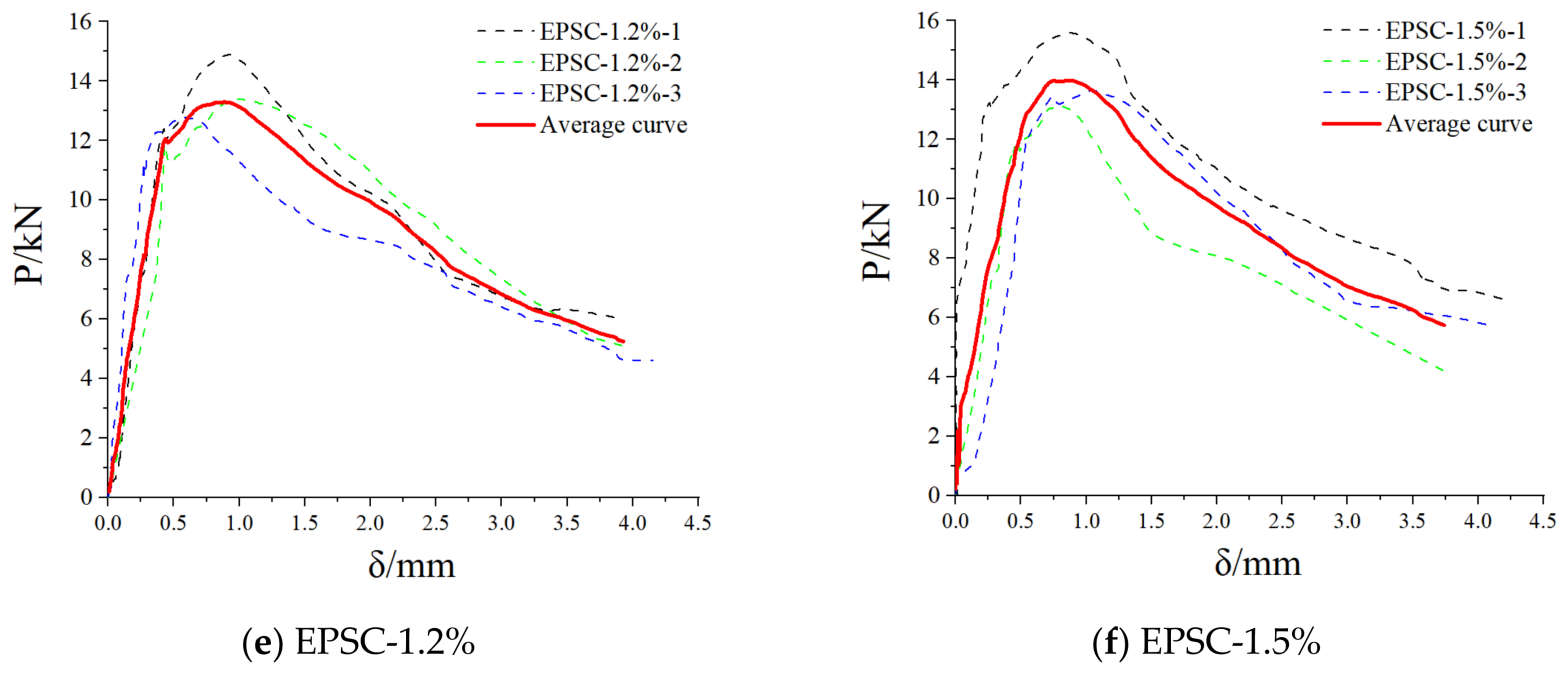
The bending load-deflection curve of EPSC.

**Figure 8:**
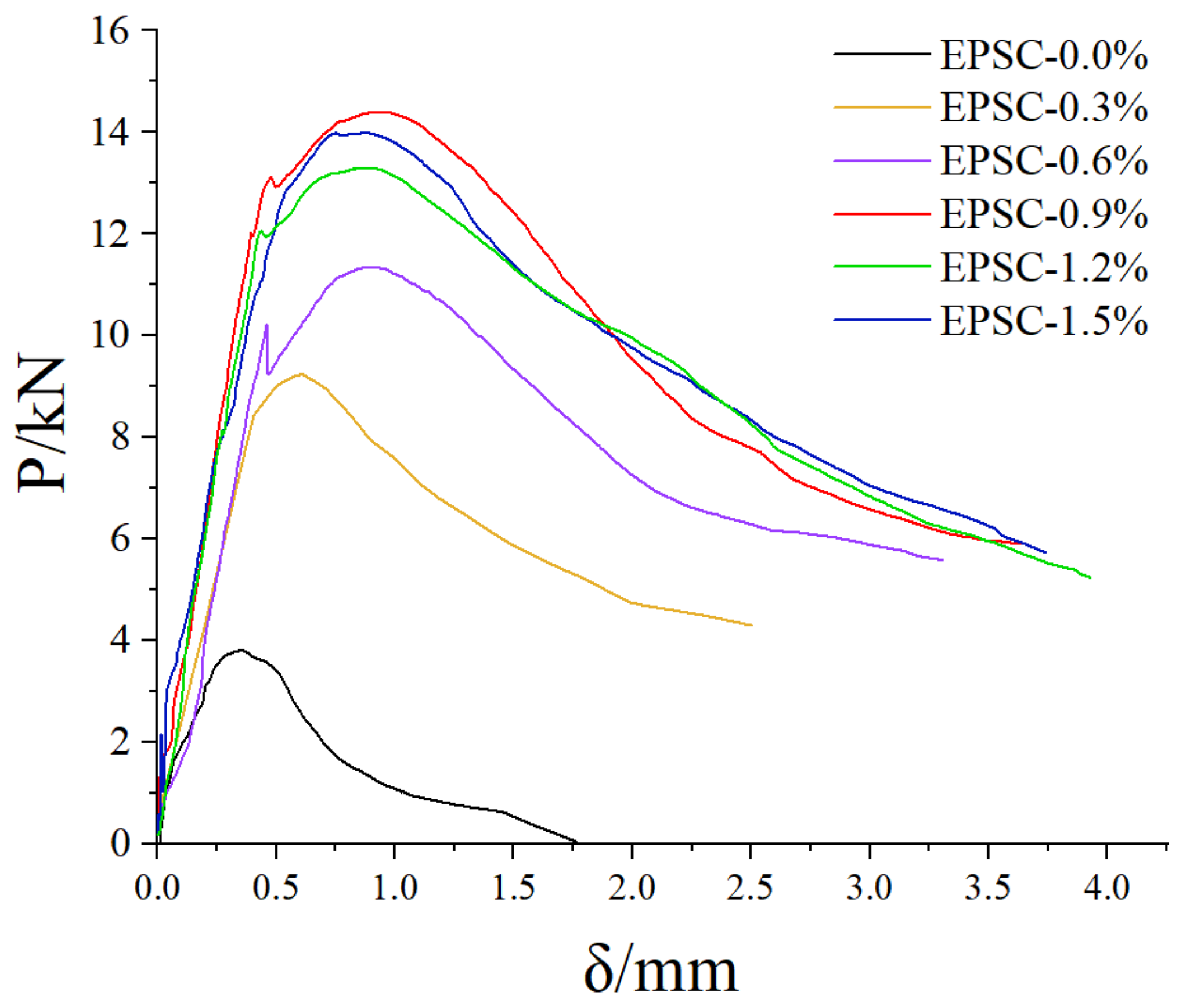
Average load-deflection curve of EPSC flexural specimens.

The bending load-deflection curves of EPSC with different HTPP fiber contents (0.0%, 0.3%, 0.6%, 0.9%, 1.2%, and 1.5%) are shown in Figure 7, and the average curves are presented in Figure 8. Based on the crack propagation process and failure modes of the specimens, the bending load-deflection curves of the specimens can be divided into the following three stages:

Stage I is the elastic deformation stage, during which the flexural load-deflection curve exhibits a linear characteristic. The addition of HTPP fibers increases the slope of the flexural load-deflection curve of EPSC, indicating that HTPP fibers effectively enhance the elastic stiffness of EPSC. However, when the HTPP fiber content exceeds 0.9%, the slope of the curve shows a decreasing trend, suggesting that excessive fibers reduce the elastic stiffness of EPSC. When the load reaches the critical value, a distinct inflection point appears in the curve of the group with HTPP fibers, and micro-cracks emerge at the bottom of the specimen, marking the end of this stage.

Stage II: Crack Propagation Stage. After concrete cracking, the stiffness decreases, and the slope of the curve gradually reduces, transitioning into a smoother curve. Crack development in this stage also consists of three phases: existing micro-cracks gradually expand, new cracks form, and then cracks progressively grow into major cracks. The load reaches its maximum value, after which it begins to decline, marking the end of Stage II. During this stage, with the incorporation of HTPP fibers, the peak load of EPSC increases significantly as the fiber content rises. When the HTPP fiber content reaches 0.9%, the peak load of EPSC attains its maximum value of 14.4 kN, compared to only 3.8 kN for specimens without fibers, representing an increase of 279.0%. In this stage, HTPP fibers effectively suppress crack propagation through strong interfacial bonding and stress transfer, demonstrating that HTPP fibers possess higher post-cracking load-bearing capacity and energy absorption capability.

Stage III is the unstable crack propagation stage. The deflection continues to increase, and the main crack further propagates. In the main crack region, fibers undergo a dual failure mechanism of pull-out and fracture, leading to a significant reduction in effective stiffness. The increasing proportion of fiber failure in the main crack region causes a sudden rise in the deformation rate and a drop in load, bringing the specimen to a critical failure state. The load-deflection curve of EPSC without HTPP fibers declines more rapidly, whereas that of EPSC with HTPP fibers exhibits a slower, gentler descending trend. The post-peak residual load increases with the increase in HTPP fiber content. The interfacial bonding advantage of HTPP fibers contributes to ensuring the post-cracking load-bearing capacity.

### 3.4. Average Residual Flexural Strength

The average residual strength (ARS) is a key indicator for evaluating flexural toughness, reflecting the material’s ability to continue bearing load after flexural failure occurs. According to the American standard ASTM C1399 [19], four-point bending tests are conducted to collect the residual flexural loads at mid-span deflections of 0.5 mm, 0.75 mm, 1.0 mm, and 1.25 mm. The arithmetic mean of these loads is then calculated using Equation (1) to obtain the average residual strength of the specimen.

(1) $$ARS=\frac{L}{bd^{2}}[\frac{P_{0.5}+P_{0.75}+P_{1.0}+P_{1.25}}{4}]$$

where L is the span of the concrete beam, and the unit is mm; b is the width of the cross-section of the concrete beam, and the unit is mm; d is the height of the cross-section of the concrete beam, in mm. P0.5, P0.75, P1.0, and P1.25 are the residual flexural strengths corresponding to mid-span deflections of 0.5 mm, 0.75 mm, 1.0 mm, and 1.25 mm, respectively, and the unit is kN.

The ARS calculation results and average values for each group of specimens are summarized in Table 3. The enhancement effect of HTPP fiber on the average residual flexural strength of EPSC exhibits significant differences. As the HTPP fiber content increases from 0.0% to 1.5%, the ARS of the specimens rises from 0.5 MPa to 4.1 MPa, and then decreases to 3.8 MPa, showing a trend of initial increase followed by a decrease. When the HTPP fiber content is 0.9%, the ARS value of EPSC reaches its maximum, which is 681.3% higher than that of EPSC without HTPP fiber. Owing to its excellent fiber-matrix interfacial bonding performance, HTPP fiber can more effectively delay the degradation of load-bearing capacity through mechanisms such as crack bridging and stress dispersion. This demonstrates the superiority of HTPP fiber in terms of flexural toughness. This trend is consistent with the toughness characteristics reflected in the earlier load-deflection curves, providing a clear basis for quantitatively evaluating the relationship between fiber content and the toughening performance of the material.

### 3.5. Evaluation of Flexural Toughness

To comprehensively evaluate the enhancement effect of HTPP fibers on the flexural toughness of EPS concrete, the flexural strength alone is insufficient to adequately characterize the ductility and energy dissipation capacity of the material during crack propagation. In addition, there are certain differences among various standards and related studies in terms of the definition, characterization parameters, and calculation methods of flexural toughness, making it difficult for a single evaluation method to fully and objectively reflect the toughening effect of fibers. In particular, the evaluation method specified in CECS 13:2009 [18] has the practical limitation that the initial cracking point is difficult to determine accurately, which in turn affects the stability and accuracy of the calculated toughness indices. Therefore, based on the load-deflection curves obtained from the experiments, the flexural toughness of HTPP fiber-reinforced EPSC was comprehensively evaluated using the Nemkumar method [20], the evaluation method reported in the literature [21], the JSCE-SF4 standard [16], and the modified PCER method [22]. Through a cross-comparative analysis of multiple methods, the variation in the flexural toughness of EPSC under different fiber contents can be systematically quantified, and the enhancement effect of HTPP fibers on the flexural behavior of the material can be revealed from different evaluation perspectives, thereby improving the reliability, rationality, and comparability of the research conclusions.

#### 3.5.1. Evaluation of Flexural Toughness by the Nemkumar Method

**Figure 9:**
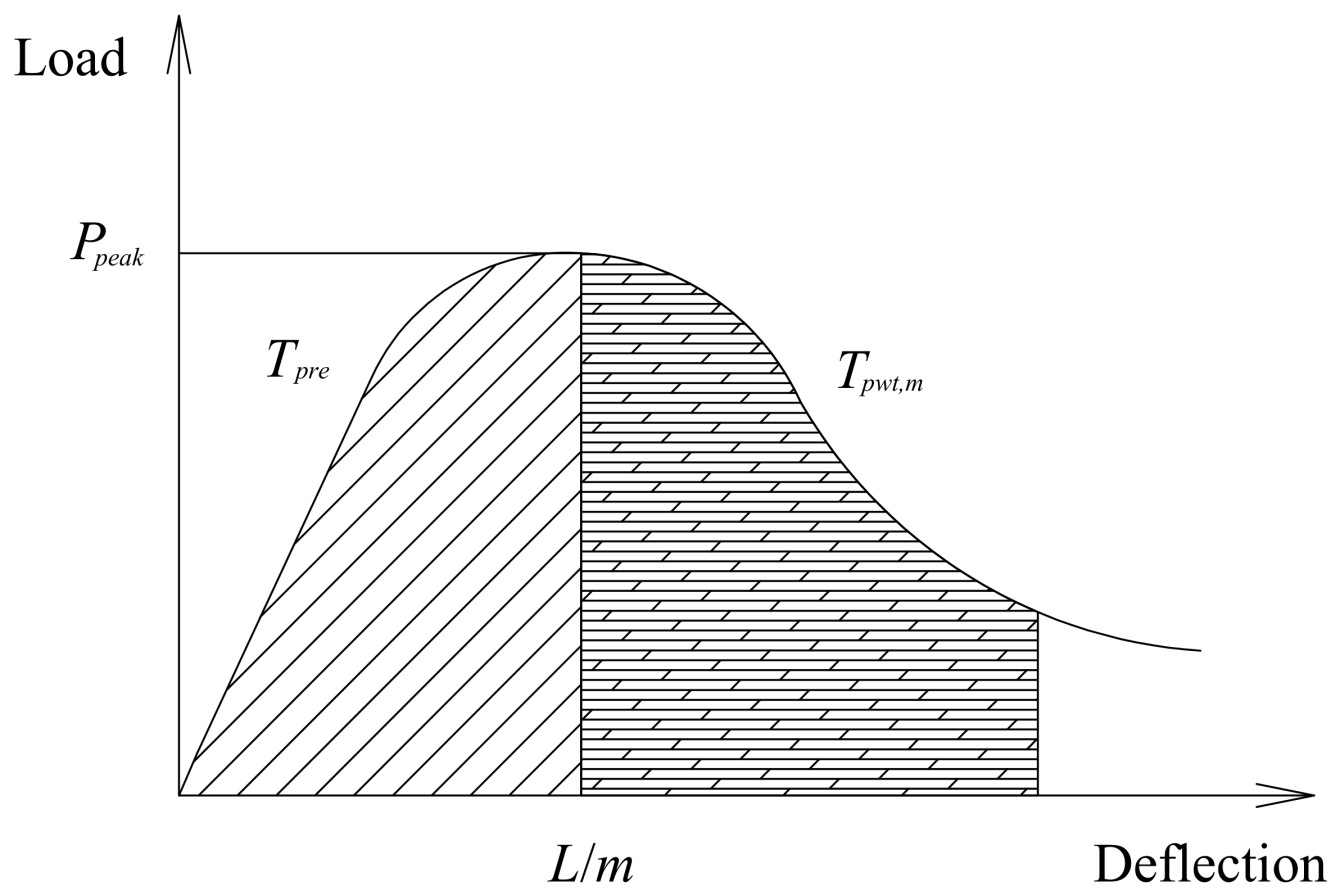
Schematic of the toughness index calculation method.

In the Nemkumar toughness index method, the peak load is taken as the reference point, and the area under the load-deflection curve is divided into a pre-peak component (T_pre_) and a post-peak component (T_pwt,m_). With a specified deflection of L/m as the variable, the method is used to evaluate the energy absorbed by fiber-reinforced concrete during deformation. A schematic diagram of the calculation procedure is shown in Figure 9.

The toughness index PCSm based on the Nemkumar method is defined by Equation (2).

(2) $$PCS_{m}=\frac{T_{\mathrm{pwt},m}L}{\left(\frac{L}{m}-\delta \right)bh^{2}},\;\left(\frac{L}{m}>\delta \right)$$

where T_pwt,m_ is the area enclosed by the post-peak portion of the load-deflection curve; L is the span of the beam (L = 300 mm in this study); δ is the deflection corresponding to the peak load; b and h are the width and height of the beam cross-section, respectively (b = h = 100 mm in this study); and L/m is the beam deflection, where m is a constant (taken as 200 in this study).

Based on the experimental results, the calculated PCS_200_ values of each specimen group are listed in Table 4. The toughness index PCS_200_ of EPSC first increased and then decreased with increasing HTPP fiber content. The maximum PCS_200_ value was obtained for EPSC containing 0.9% HTPP fibers. Compared with EPS concrete without HTPP fibers, the addition of HTPP fibers increased PCS_200_ by up to 824.31%.

#### 3.5.2. Literature-Based Evaluation of Flexural Toughness

Reference [21] proposed the use of the initial flexural toughness ratio R_e,p_ and the residual flexural toughness ratio R_e,k_ to characterize the flexural toughness of specimens before and after reaching the peak deflection, respectively. This method does not depend on the initial cracking deflection. Among these parameters, the flexural toughness index before the peak deflection is expressed by R_e,p_, which is calculated using Equation (3).

(3) $$R_{e,p}=\frac{f_{e,p}}{f_{ftm}}$$

where *f*_ftm_ is the flexural strength (MPa), and *f*_e,p_ is the equivalent initial flexural strength (MPa), which is calculated by Equation (4):

(4) $$f_{e,p}=\frac{\Omega_{p}L}{bh^{2}\delta_{p}}$$

where Ω_p_ is the area under the load-deflection curve before the peak deflection δp (N·mm), and b and h are the width and height of the specimen, respectively.

The flexural toughness index after the peak deflection is represented by the residual flexural toughness ratio Re,k, which is calculated using Equation (5):

(5) $$R_{e,k}=\frac{f_{e,k}}{f_{ftm}}$$

where *f*_e,k_ is the equivalent residual flexural strength (MPa), which is calculated by Equation (6):

(6) $$f_{e,k}=\frac{\Omega_{R,k}L}{bh^{2}\delta_{R,k}}$$

where Ω_R,k_ is the area enclosed by the load-deflection curve from δp to δk (N·mm), and δk is the calculated mid-span deflection, defined as L/k. In this study, k = 150. The beam span L was 300 mm, and both b and h were 100 mm.

The calculated results are presented in Table 5 and Table 6. It can be seen that the flexural toughness indices of EPSC were significantly improved after the incorporation of HTPP fibers. With increasing HTPP fiber content, the relevant flexural toughness indices showed a trend of first increasing and then decreasing. When the fiber content was 0.9%, these values reached their maximum, increasing by 11.3% and 225.05%, respectively. As the fiber content continued to increase, these values decreased again. The results calculated by this method were similar to those obtained using the Nemkumar method. This method indicates that HTPP fibers have a more pronounced effect on improving the post-peak flexural toughness.

#### 3.5.3. JSCE-SF4 Method for Flexural Toughness Evaluation

**Figure 10:**
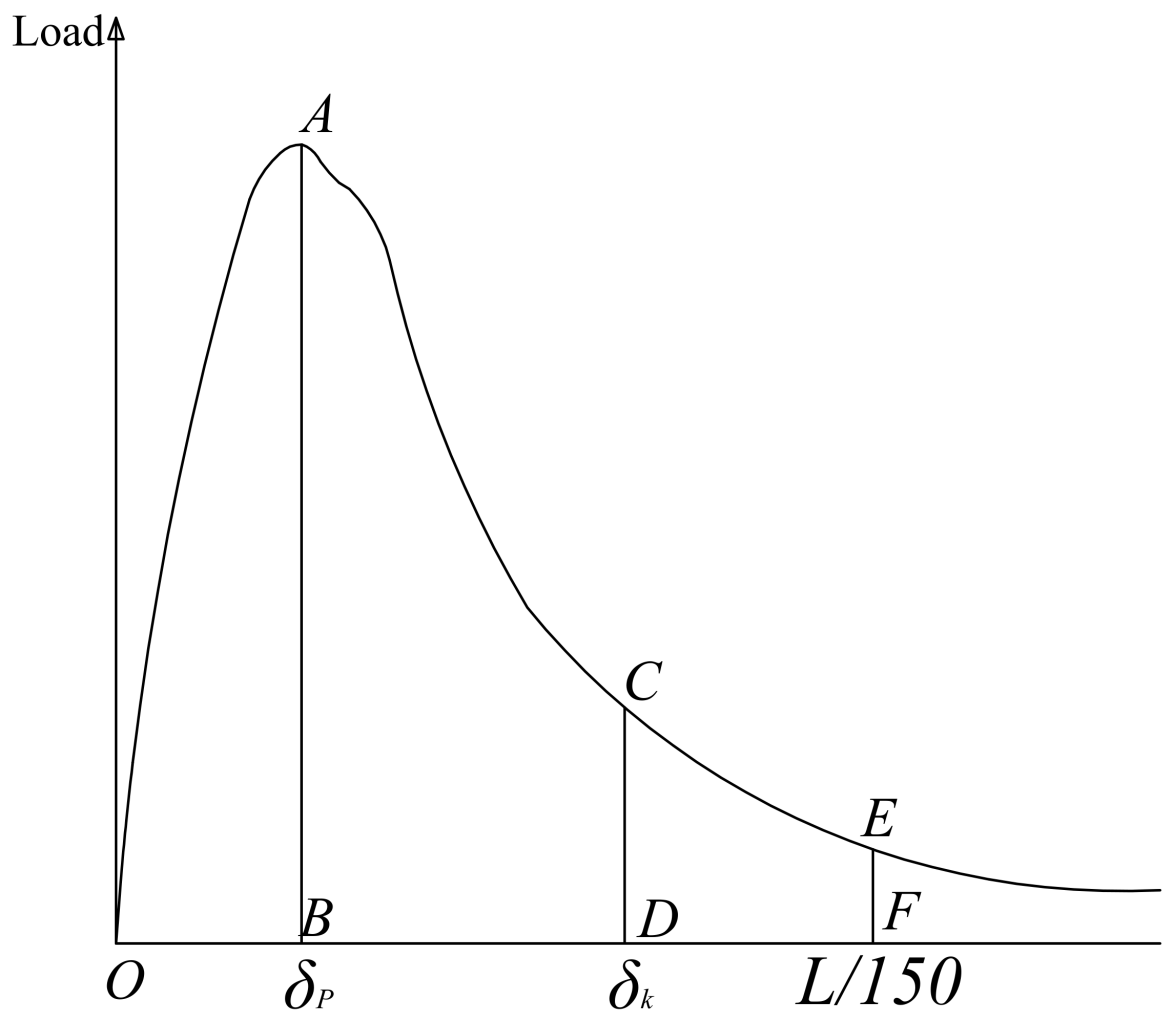
Definition of the flexural toughness index.

The JSCE-SF4 standard evaluates the flexural toughness of fiber-reinforced concrete using the equivalent flexural strength *f*_e_ (MPa). The Chinese standard CECS 13:2009 [18] adopts this evaluation approach with reference to the JSCE-SF4 standard. The equivalent flexural strength *f*_e_ is calculated using Equation (7).

(7) $$f_{e}=\frac{\Omega_{k}L}{bh^{2}\delta_{k}}$$

where Ω_*k*_ is the area under the load-deflection curve of the flexural specimen when the calculated mid-span deflection reaches L/k (N·mm). In the JSCE-SF4 standard, k = 150, and Ω_*k*_ corresponds to the area enclosed by OAEF in Figure 10. δ_k_ is the deflection value at the calculated mid-span deflection of L/k (mm), where k = 150; L is the span between the supports (mm); and b and h are the width (mm) and height (mm) of the specimen cross-section, respectively.

This evaluation method has the advantages of a clear concept, simple calculation, and independence from the determination of the initial cracking point. In addition, the area of the unstable segment of the curve is relatively small compared with the area under the curve at a calculated mid-span deflection of L/150; therefore, the unstable segment of the load-deflection curve has little influence on the equivalent flexural strength. The calculated *f*_e_ values obtained in this study are listed in Table 7. As shown in Table 7, with increasing HTPP fiber content, *f*_e_ first increased and then decreased. The maximum value was obtained at a fiber content of 0.9%, representing an increase of 699.3% compared with EPSC without fibers.

#### 3.5.4. Evaluation of Flexural Toughness by the Modified PCER Method

**Figure 11:**
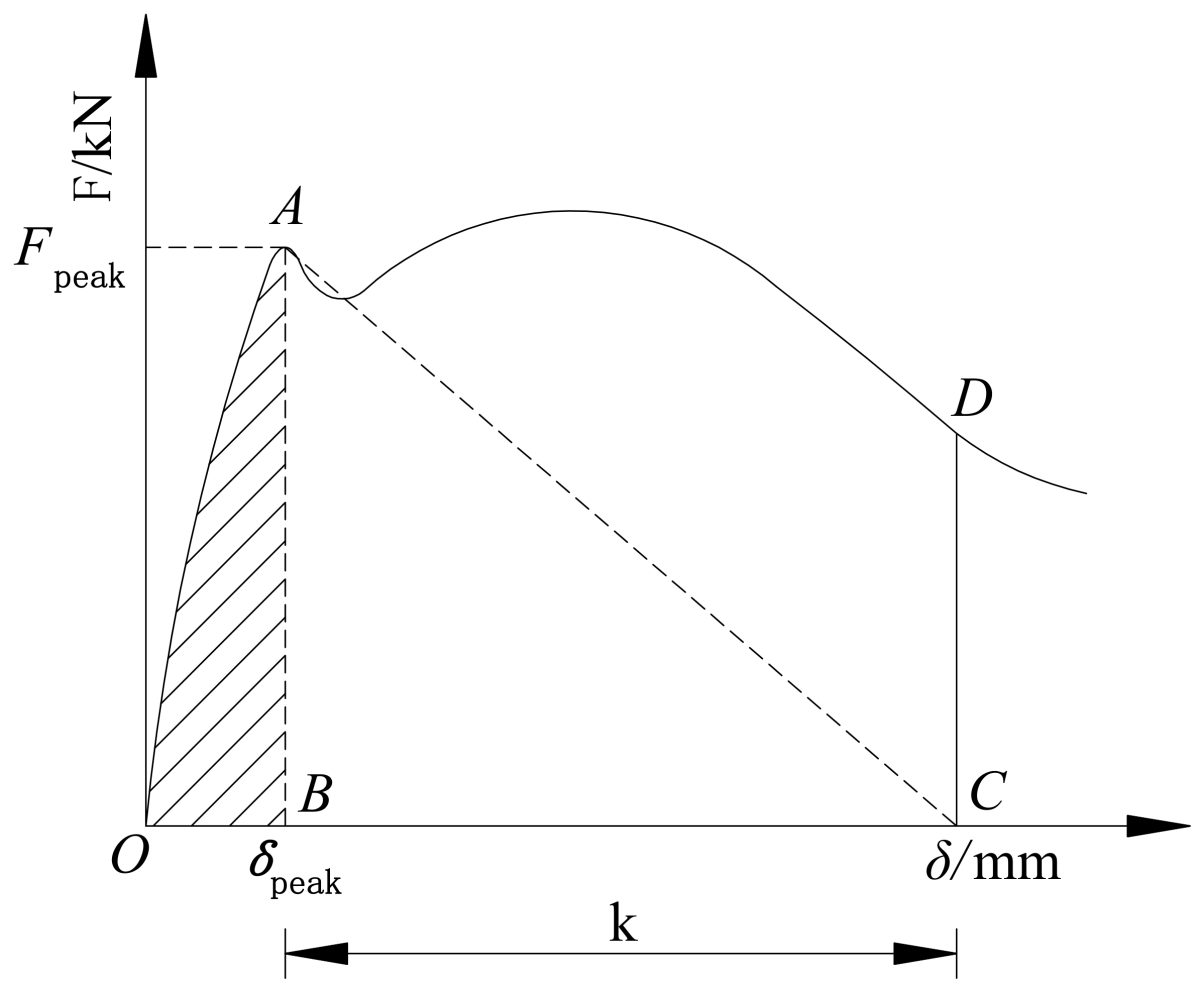
Schematic diagram of the modified PCER method for flexural toughness evaluation.

Professor Jianguo Han of Tsinghua University [22], based on a comprehensive review of several domestic and international toughness evaluation methods for concrete materials, proposed a new method for evaluating the flexural toughness of concrete materials, termed the PCER method; namely, the post-peak energy ratio method. This method is based on the bilinear model of an ideal concrete material, and evaluates concrete toughness by calculating the ratio of the area under the measured load-deflection curve after the peak load to the corresponding post-peak area of the ideal bilinear model. A schematic illustration of the calculation is shown in Figure 11, and the calculation formula is given in Equation (8).

(8) $$PCER=\frac{\Omega_{post}}{0.5F_{peak}k}$$

where PCER is the post-peak energy ratio; Ω_post_ is the measured post-peak area, namely the area of region ABCD in the figure (N·mm); F_peak_ is the peak load (N); and k is a prescribed value, with a recommended range of 0.1–10 mm.

Although this method can reflect the flexural toughness level of concrete, it is somewhat inadequate because it does not make use of the data from the load-displacement curve before the peak load. With reference to CECS 13:2009, the initial flexural toughness ratio Re,p was introduced to characterize the flexural toughness of concrete before reaching the peak deflection. The difference is that the denominator adopts the peak strength of concrete instead of the initial cracking strength. Compared with the initial cracking strength, the peak strength is easier to determine and makes the calculation results more accurate. Therefore, Re,p was used to characterize the flexural toughness before the peak strength was reached, and its calculation formula is given in Equation (9).

(9) $$R_{e,p}=f_{e,p}/f_{peak}$$

where *f*_peak_ is the flexural strength of concrete (MPa), and *f*_e,p_ is the equivalent initial flexural strength of concrete, which is calculated by Equation (10).

(10) $$f_{e,p}=\frac{\Omega_{peak}L}{bh^{2}\delta_{peak}}$$

where Ωp is the area under the load-deflection curve before the peak deflection δ_peak_ (N·mm); namely, the area enclosed by OAB in Figure 10.

A schematic diagram of the modified PCER method for flexural toughness evaluation is shown in Figure 11. Using the modified PCER method to characterize the flexural toughness of concrete, k was taken as 2 in Figure 11, and the calculation results are listed in Table 8.

The modified PCER method is simple in calculation, avoids the inconvenience associated with determining the initial cracking point, and is applicable to cases where the deflection corresponding to the peak load is relatively large. By characterizing the toughness before and after the peak load separately, it can effectively evaluate the flexural toughness over the entire load-deflection curve. As shown in Table 8, both PCER and R_e,p_ reached their maximum values when the HTPP fiber content was 0.9%. Moreover, with increasing HTPP fiber content, both PCER and R_e,p_ of EPSC first increased and then decreased. Compared with EPSC without HTPP fibers, the maximum increases in PCER and R_e,p_ were 241.2% and 9.0%, respectively. The toughness of EPSC is closely related to the fiber content, and an appropriate HTPP fiber dosage can optimize the toughness of EPSC.

#### 3.5.5. Evaluation of Flexural Toughness Methods

The above four flexural toughness evaluation methods can all effectively characterize the enhancement effect of HTPP fibers on the flexural toughness of EPSC, and the conclusions obtained are in good agreement. Although these methods differ in calculation principles and forms of characterization, they all indicate that the incorporation of HTPP fibers significantly improves the flexural toughness of EPSC. All toughness indices first increase and then decrease with increasing fiber content, reaching the optimum value at a fiber content of 0.9%. This demonstrates that HTPP fibers have a pronounced toughening effect on EPSC, and that an appropriate fiber dosage can effectively improve the flexural performance of the material.

In terms of applicability, the Nemkumar method, the literature-based method, the JSCE-SF4 method, and the modified PCER method can all reflect the variation in the flexural toughness of EPSC from different perspectives. Among them, the Nemkumar method and the modified PCER method provide a more direct characterization of post-peak toughness behavior, clearly reflecting the enhancement effect of HTPP fibers on post-cracking load-carrying capacity and post-peak energy dissipation. The literature-based method can separately characterize the toughness before and after the peak point, which is helpful for a more detailed analysis of the influence of fibers on flexural behavior at different loading stages. The JSCE-SF4 method is simple to calculate and convenient for rapid evaluation in engineering applications; however, because the specified deflection value is relatively large, the resulting equivalent flexural strength is essentially the average strength of the specimen within the target deflection range, making it difficult to reflect in detail the evolution of mechanical behavior before and after cracking. By contrast, the Nemkumar method only uses the area under the post-peak portion of the load-deflection curve and the corresponding deflection value, and therefore gives insufficient consideration to the pre-peak stage.

Although the four evaluation methods generally yield consistent trends regarding the effect of fiber content, the absolute values and relative improvements of the resulting indices differ to some extent. These differences are mainly attributable to variations in the integration intervals, reference deflections, and normalization parameters adopted by the respective methods. The Nemkumar method focuses on the post-peak energy absorption capacity within a specified deflection range and is therefore relatively sensitive to changes in residual load-carrying capacity induced by fiber bridging. The JSCE-SF4 method uses the total area under the load-deflection curve up to a prescribed deflection, thereby reflecting the average equivalent load-carrying capacity within that deflection range. In contrast, the modified PCER method characterizes post-peak energy retention through the ratio of the measured post-peak energy to that of an idealized bilinear model. Therefore, the numerical values and relative improvements obtained using different methods should not be compared directly; nevertheless, their trends with increasing fiber content can be used for cross-validation.

In summary, the combined use of multiple evaluation methods not only enables cross-validation among the different results, thereby improving the reliability and objectivity of the conclusions, but also provides a more comprehensive understanding of the enhancement effect of HTPP fibers on the flexural performance of EPSC.

### 3.6. Comprehensive Analysis of the Optimum Fiber Content

In this study, the designation of 0.9% as the optimum fiber content refers specifically to the finding that 0.9 vol.% HTPP fiber provided the best overall performance within the investigated range of 0–1.5 vol.%. This does not imply that this dosage is universally applicable to all EPS concrete mixtures or engineering conditions.

At a fiber volume fraction of 0.9%, the compressive strength, flexural strength, peak load, average residual flexural strength, PCS200, residual flexural toughness ratio, equivalent flexural strength, and PCER of EPS concrete all reached or approached their maximum values. These indices reflect matrix strength, pre-cracking load-carrying capacity, post-cracking residual load-carrying capacity, and energy absorption capacity, respectively. Their generally consistent trends indicate that a relatively favorable balance between fiber reinforcement and matrix integrity was achieved at a fiber content of 0.9%.

When the fiber content was further increased to 1.2% and 1.5%, the greater number of fibers could potentially provide more crack-bridging sites. However, excessive fiber incorporation may reduce the workability of the fresh mixture, increase fiber–fiber interference, promote local fiber agglomeration, and increase the air content, thereby weakening the continuity of the cementitious matrix and reducing the effectiveness of fiber anchorage. Consequently, further increases in fiber content did not produce corresponding improvements in performance, and some indices instead exhibited a decreasing trend.

## 4. Microscopic Mechanism of HTPP Fiber-Reinforced EPSC

**Figure 12:**
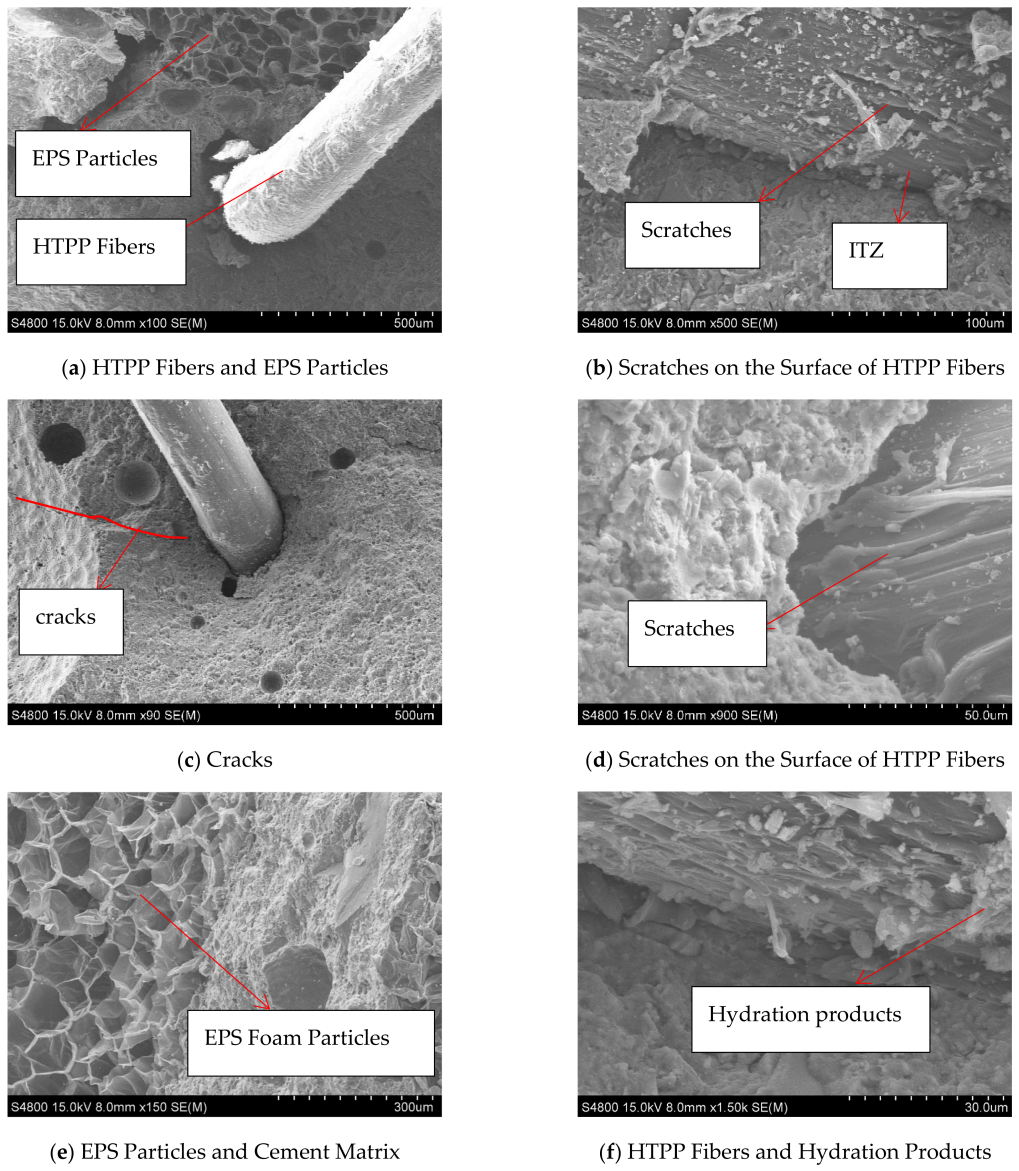
Microstructure of EPSC.

The microstructure of EPSC specimens is shown in Figure 12. It can be observed that HTPP fibers can be dispersed relatively uniformly in the matrix after mixing, and mainly exert a reinforcing effect in the form of single bundles. During the failure process, HTPP fibers exhibit various failure modes including fiber pull-out, fiber breakage, and pull-out followed by breakage, indicating that the fibers can continuously participate in crack propagation control and energy dissipation under loading.

In terms of crack resistance, HTPP fibers exert a significant inhibitory effect on crack propagation. As shown in Figure 12c, cracks are blocked by fibers during propagation, demonstrating that the fibers induce crack path deflection. HTPP fibers bridge across both sides of cracks, effectively restraining further crack development and optimizing the stress distribution in the crack zone, thereby improving the overall strength and post-peak deformation capacity of the material.

Regarding interfacial bonding characteristics, the Interface Transition Zone (ITZ) between HTPP fibers and the EPSC matrix is relatively dense with good interfacial adhesion, as illustrated in Figure 12a. The “voids” observed between fibers and the matrix at some locations are mainly debonding traces formed during tensile pull-out of the fibers, rather than caused by loose original interfaces. This indicates a strong bonding performance between HTPP fibers and the matrix. During crack propagation, the fibers dissipate energy input by external loads through interfacial slip, debonding, and pull-out, thus delaying the initiation and propagation of initial microcracks.

From the perspective of fiber surface morphology and hydration product adhesion, numerous hydration products are attached to the surface of HTPP fibers with tight bonding. The rough surface morphology readily provides nucleation sites for hydration products. Moreover, the introduction of hydrophilic groups through fiber grafting facilitates the formation and deposition of hydration products such as C-S-H gel and Ca(OH)_2_ on the fiber surface, further enhancing the interfacial bonding between fibers and the matrix, as shown in Figure 12f. A large number of scratches and attached matrix residues can be seen on the fiber surface after failure, as presented in Figure 12b,d,f. This reveals that the fibers undergo significant frictional slip, debonding, and pull-out during continuous crack propagation, and further verifies the strong interfacial interlocking between HTPP fibers and the EPSC matrix.

Taken together, the observed microstructural features reveal fiber bridging, pull-out, rupture, and hydration products adhering to the fiber surfaces in the vicinity of cracks. These features are consistent with the improvements in post-peak load-carrying capacity and energy absorption observed in the macroscopic tests, and thus provide qualitative support for the proposed toughening mechanism of HTPP fibers. It should be noted, however, that the SEM investigation in this study was primarily intended to characterize representative microstructural features, and no systematic quantitative analysis of the three-dimensional fiber distribution or interfacial parameters was conducted. Therefore, the quantitative relationships among fiber dispersion, orientation characteristics, interfacial transition-zone width, and macroscopic toughness indices require further validation through image-based statistical analysis, single-fiber pull-out tests, and X-ray computed tomography.

## 5. Preliminary Estimation of Support Spacing for EPSC Exterior Wall Panels Based on Residual Flexural Strength

To investigate the effect of different HTPP fiber contents on the flexural performance of EPSC exterior wall panels under actual wind pressure loads, a wind-resistant design analysis of the panels was conducted based on the results of material flexural performance tests and a computational model for support spacing under wind loads.

Referring to the calculation method for rectangular plates under uniformly distributed loads [23], the stress distribution of EPSC wall panels under wind loads was analyzed. Drawing on the current standard T/CECS 1216-2022 [24], the flexural performance of EPSC exterior wall panels under wind loads can be evaluated based on the long-side span between support points, using the following formula:

(11) $$\gamma_{0}\sigma \leq \frac{f_{Mk}}{\gamma_{m}}$$

where $\gamma_{0}$ is the member importance coefficient, $\gamma_{0}\geq 1.0$, which is not considered for seismic design; σ is the design value of the sectional stress of the EPSC exterior wall panel under the basic combination, MPa; $f_{Mk}$ is the characteristic value of the flexural stress of the EPSC exterior wall panel face, MPa; and $\gamma_{m}$ is the partial safety factor for concrete, taken as 1.4.

To ensure that the exterior wall panel retains its load-bearing capacity after cracking and to increase the safety margin, the characteristic value of flexural stress $f_{Mk}$ is taken as the average residual bending strength (ARS) of the EPSC. For a four-point-supported exterior wall panel, the formula for calculating the characteristic value of sectional stress under wind loads is as follows:

(12) $$\sigma_{k}=\frac{6mq_{k}l_{y}^{2}}{d^{2}}$$

where $\sigma_{k}$ is the characteristic value of the sectional stress in the exterior wall panel under wind loads, MPa; $q_{k}$ is the characteristic value of wind load, MPa; $l_{y}$ is the long-side span between support points of the exterior wall panel, mm; *d* is the thickness of the exterior wall panel, mm; and *m* is the bending moment coefficient for a four-point-supported exterior wall panel, taken as 0.154 for a square panel.

**Figure 13:**
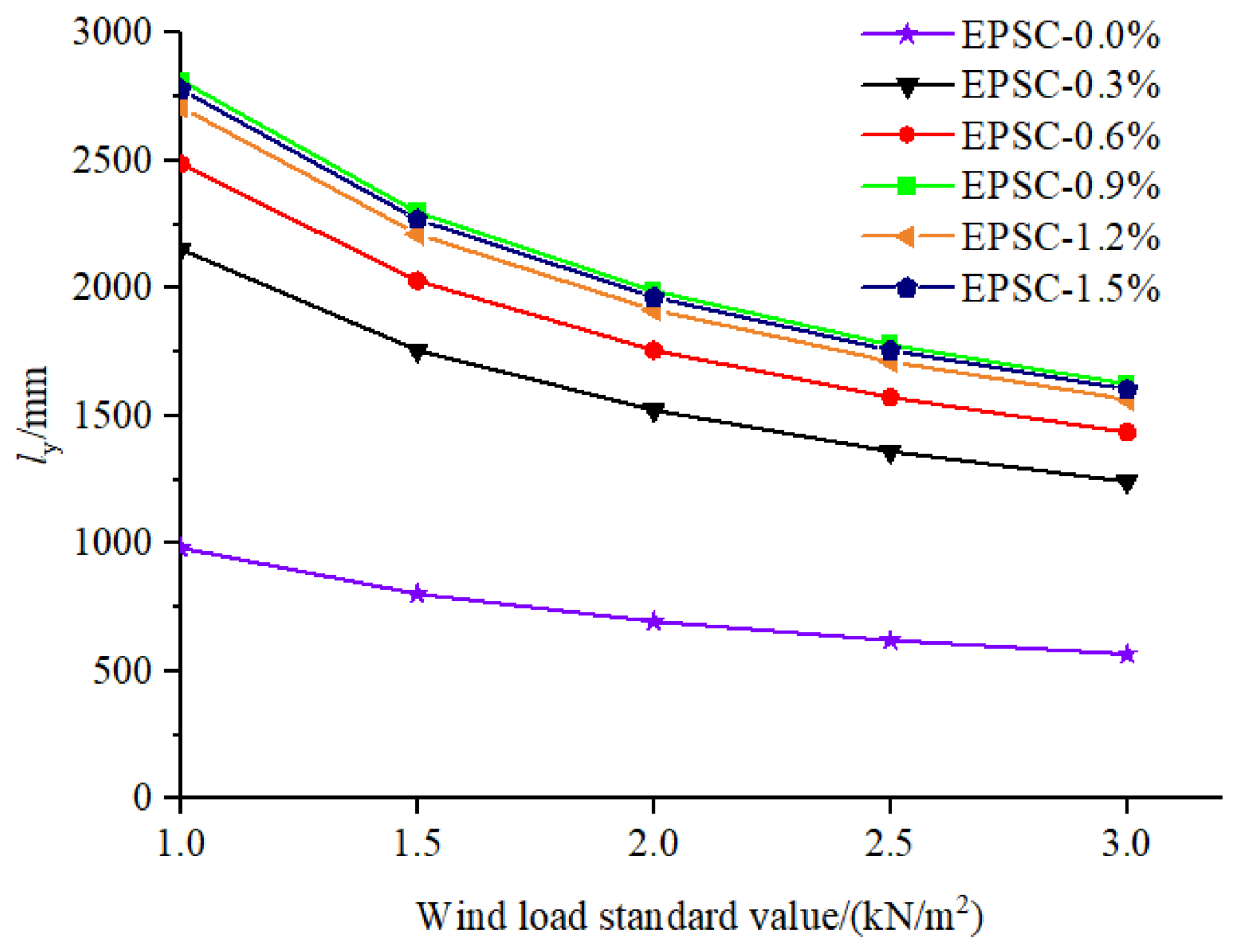
Relationship curve between support spacing of wall panels and wind load.

Referring to the calculation methods for wind loads on building envelopes, the average ARS value of the EPSC test specimens was used as the post-cracking flexural resistance control parameter. A simplified model approximating a square plate with four-side support was adopted to perform wind resistance verification on 50 mm EPSC exterior wall panels. The maximum spacing of support points for EPSC exterior wall panels under different characteristic wind load values was calculated using Equations (11) and (12), with the results shown in Table 9 and Figure 13. The calculation results indicate that the maximum spacing between support points of the exterior wall panels increases with higher ARS values. The wind resistance capacity of the exterior wall panels is optimal when the HTPP fiber content is 0.9%, suggesting that the appropriate addition of HTPP fibers can significantly improve the post-cracking load-bearing capacity and wind resistance performance of EPSC exterior wall panels. Further analysis indicates that, with the addition of HTPP fibers, EPSC exterior wall panels can achieve greater unsupported spans and exhibit superior post-cracking load-bearing capacity and deformation adaptability. This facilitates resistance to wind loads and control of crack propagation, thereby ensuring the integrity and safety of the building envelope. From an engineering application perspective, HTPP fiber-reinforced EPSC wall panels help reduce the number of backing steel frames required per unit area, simplify structural joints, lower material and construction costs, and improve installation efficiency. The above results indicate that HTPP fiber-reinforced EPSC wall panels can meet the application requirements of larger panel sizes and fewer support points, exhibiting good engineering applicability and economic efficiency, and can provide a basis for the optimized design of lightweight, high-performance enclosure structures.

## 6. Conclusions

This study systematically investigated the effect of HTPP fiber on the flexural toughness of EPS concrete. Based on four-point bending tests, the main conclusions are as follows:

(1) HTPP fiber can significantly improve the flexural toughness of EPS concrete. The specimen exhibits the strongest post-peak load-bearing capacity and the optimal toughening effect at a fiber content of 0.9%.

(2) The average residual flexural strength (ARS) first increases and then decreases with the increase of HTPP fiber content, reaching its maximum at 0.9%. Compared with plain EPS concrete, ARS is increased by 681.3%.

(3) Different flexural toughness evaluation methods can well characterize the toughening regularity of HTPP fiber on EPSC. Among them, the Nemkumar method and the modified PCER method are more intuitive and applicable in characterizing the post-cracking load-bearing capacity of the material.

(4) The toughening effect of HTPP fiber on EPSC is mainly reflected in crack bridging, deflecting crack propagation paths, and continuously dissipating energy under external loads. The processes of fiber pull-out, debonding, and frictional slip further enhance the post-cracking load-bearing capacity and deformability of the material.

(5) The incorporation of HTPP fibers significantly improved the wind resistance and post-cracking behavior of EPSC exterior wall panels, effectively enhancing their unsupported span capacity and feasibility for engineering applications.

## Figures and Tables

**Table 1:** Parameters of HTPP fibers.

Name	Density/(kg·m^−3^)	Diameter/mm	Length/mm	Elastic Modulus/MPa	Tensile Strength/MPa	Fracture Strength/MPa	Elongation at Break/%
HTPP Fibers	900	0.18	12	8–10	800–1200	750	14

**Table 2:** Mix proportions of EPSC (per 1 m^3^).

EPS Concrete No.	Cement/kg	Fly Ash/kg	EPS Particles/L	Water/kg	Water Reducer/kg	HTPP Fiber Volume Content/%
EPSC-0.0%	370	370	580	272	2.0	0%
EPSC-0.3%	370	370	580	272	2.0	0%
EPSC-0.6%	370	370	580	272	2.0	0.6%
EPSC-0.9%	370	370	580	272	2.0	0.9%
EPSC-1.2%	370	370	580	272	2.0	1.2%
EPSC-1.5%	370	370	580	272	2.0	1.5%

**Table 3:** ABS values of EPSC specimens in each group.

Specimen Number	$P_{0.5}$/kN	$P_{0.75}$/kN	$P_{1.0}$/kN	$P_{1.25}$/kN	ARS/MPa	ARS Average/MPa
EPSC-0.0%-1	4.216	2.030	1.315	1.026	0.644	0.5
EPSC-0.0%-2	2.765	1.339	0.683	0.351	0.385	
EPSC-0.0%-3	3.391	1.809	1.251	0.933	0.553	
EPSC-0.3%-1	8.851	8.901	8.226	6.854	2.462	2.4
EPSC-0.3%-2	10.501	9.748	7.550	6.525	2.574	
EPSC-0.3%-3	7.708	7.846	7.051	6.775	2.203	
EPSC-0.6%-1	10.662	12.506	12.486	11.869	3.564	3.2
EPSC-0.6%-2	7.836	10.017	10.772	10.239	2.914	
EPSC-0.6%-3	9.945	10.840	10.304	9.161	3.018	
EPSC-0.9%-1	11.894	12.747	13.054	12.967	3.799	4.1
EPSC-0.9%-2	14.176	15.082	15.303	14.375	4.420	
EPSC-0.9%-3	12.750	14.622	14.680	13.345	4.154	
EPSC-1.2%-1	12.431	14.475	14.662	13.411	4.123	3.8
EPSC-1.2%-2	11.331	12.687	13.404	13.119	3.790	
EPSC-1.2%-3	12.629	12.417	11.258	10.223	3.490	
EPSC-1.5%-1	14.351	15.433	15.397	14.510	4.477	4.0
EPSC-1.5%-2	11.677	13.071	12.369	10.577	3.577	
EPSC-1.5%-3	10.482	13.460	13.605	13.351	3.817	

**Table 4:** Calculated results of PCS_m_.

No.	δ	T_pwt,m_	PCS_m_
EPSC-0.0%	0.3520	1.8809	0.4915
EPSC-0.3%	0.6081	6.2396	2.0990
EPSC-0.6%	0.8999	6.2831	3.1409
EPSC-0.9%	0.9258	7.7599	4.0543
EPSC-1.2%	0.8846	7.5864	3.7001
EPSC-1.5%	0.8716	8.0991	3.8658

**Table 5:** $R_{e,p}$ Results.

No.	$\delta_{p}$ (mm)	$\Omega_{p}$ (N·mm)	$f_{e,p}$ (MPa)	$f_{ftm}$ (MPa)	$R_{e,p}$
EPSC-0.0%	0.3520	877.2236	0.7476	1.153	0.648
EPSC-0.3%	0.6081	3303.1904	1.6296	2.790	0.584
EPSC-0.6%	0.8999	6844.5556	2.2818	3.651	0.625
EPSC-0.9%	0.9258	9675.3466	3.1352	4.349	0.721
EPSC-1.2%	0.8846	8474.5618	2.8740	4.107	0.700
EPSC-1.5%	0.8716	8654.4331	2.9789	4.192	0.711

**Table 6:** $R_{e,k}$ Results.

No.	$\delta_{R\cdot k}$ (mm)	$\Omega_{R\cdot k}$ (N·mm)	$f_{e,k}$ (MPa)	$R_{e,k}$
EPSC-0.0%	1.6480	2879.6318	0.5242	0.4547
EPSC-0.3%	1.3919	12,866.1583	2.7731	0.9939
EPSC-0.6%	1.1001	17,346.2393	4.7304	1.2956
EPSC-0.9%	1.0742	23,015.3044	6.4277	1.4780
EPSC-1.2%	1.1154	21,444.0364	5.7676	1.4043
EPSC-1.5%	1.1284	22,031.5565	5.8574	1.3973

**Table 7:** $f_{e}$ calculation results.

No.	T_b_ (N·mm)	$f_{e}$ (MPa)
EPSC-0.0%	2879.6318	0.4319
EPSC-0.3%	12,866.1583	1.9299
EPSC-0.6%	17,346.2393	2.6019
EPSC-0.9%	23,015.3044	3.4523
EPSC-1.2%	21,444.0364	3.2166
EPSC-1.5%	22,031.5565	3.3047

$\delta_{tb}$ = 2, L = 300 mm, b = 100 mm, h = 100 mm.

**Table 8:** Calculation results of the modified PCER flexural toughness indices.

No.	*F*_peak_/kN	*f*_peak_/MPa	R_e,p_	PCER
EPSC-0.0%	3.81	1.14	0.67	0.51
EPSC-0.3%	12.46	3.74	0.50	1.15
EPSC-0.6%	11.35	3.41	0.67	1.43
EPSC-0.9%	14.41	4.32	0.73	1.74
EPSC-1.2%	13.30	3.99	0.72	1.53
EPSC-1.5%	13.99	4.20	0.71	1.49

**Table 9:** Maximum spacing of support points for exterior wall panels under different wind loads (mm).

Specimen Number	Characteristic Wind Load Values/(kN/m^2^)				
	1.0	1.5	2.0	2.5	3.0
EPSC-0.0%	983	803	695	622	568
EPSC-0.3%	2154	1758	1523	1362	1243
EPSC-0.6%	2487	2030	1758	1573	1436
EPSC-0.9%	2815	2298	1990	1780	1625
EPSC-1.2%	2710	2213	1916	1714	1565
EPSC-1.5%	2780	2270	1966	1758	1605

## Data Availability

The original contributions presented in this study are included in the article. Further inquiries can be directed to the corresponding authors.

## Nomenclature

HTPP	High-Toughness Polypropylene
EPS	Expanded polystyrene
SEM	Scanning electron microscopy
ARS	Residual flexural strength
PCE	Polycarboxylate superplasticizer
EPSC	Expanded polystyrene concrete
PP	Polypropylene
ITZ	Interface Transition Zone
